# Gingival crevicular fluid as a periodontal diagnostic indicator- I: Host derived enzymes and tissue breakdown products

**Published:** 2012-12-25

**Authors:** G Gupta

**Affiliations:** Department of Periodontics, Institute of Dental Studies and Technologies, Modinagar

**Keywords:** Biomarkers, Host derived enzymes, Tissue breakdown products

## Abstract

Researchers involved in the delivery of periodontal therapy are currently investigating the possible use of oral fluids in the diagnosis of oral diseases and drug development. Substantial improvements have been made in the understanding of the mediators implicated on the initiation, pathogenesis, and progression of periodontitis. This review will analyze the mechanisms involved in the breakdown of periodontal supporting tissues during chronic periodontitis and highlights the potential array of biomarkers present in gingival crevicular fluid (GCF), which may relate to existing or predicted tissue regions undergoing metabolic change.

## Introduction

Periodontitis is a set of inflammatory diseases affecting the periodontium, i.e., the tissues that surround and support the teeth. Periodontitis involves progressive loss of the alveolar bone around the teeth, and if left untreated, can lead to the loosening and subsequent loss of teeth. It is caused by microorganisms that adhere to and grow on the tooth's surfaces, along with an overly aggressive immune response against these microorganisms [**1**].

 Damage to the periodontal tissue is usually detected by means of periodontal probing, which shows loss of attachment of the tooth, or by radiographs that detect alveolar bone loss. These methods also evaluate the damage caused by previous destruction episodes, resulting in a retrospective diagnosis [**2**].

 Accurate detection of periodontal sites exhibiting disease progression or those at risk of future deterioration has proven difficult. The development of a test for most mediators associated with the anatomic events of periodontitis may serve as a useful method for identifying and predicting future progression [**3**]. Of the 3 fluids found within the oral cavity—gingival crevicular fluid (GCF), serum, and total saliva—the first two have been the focus of the most research in recent years. Due to the non-invasive and simple nature of their collection, analysis of saliva and GCF may be especially beneficial in the determination of current periodontal status and a means of monitoring response to treatment [**4,5**].

### GCF-as a diagnostic marker

GCF is an inflammatory exudate that seeps into gingival crevices or periodontal pockets around teeth with inflamed gingival [**6**]. It is composed of serum and locally generated materials such as tissue breakdown products, inflammatory mediators, and antibodies directed against dental plaque bacteria. The composition of the GCF is the result of the interplay between the bacterial biofilm adherent to the tooth surfaces and the cells of the periodontal tissues. The collection of GCF is a minimally invasive procedure and the analysis of specific constituents in the GCF provides a quantitative biochemical indicator for the evaluation of the local cellular metabolism that reflects a person’s periodontal health status [**7**]. Since GCF is an inflammatory exudate that reflects ongoing events in the periodontal tissues that produce it, an extensive search has been made for GCF components that might serve as potential diagnostic or prognostic markers for the progression of periodontitis [**8**]. 

Curtis et al. [**9**] stated that "markers of disease" might encompass three separate categories: 

 1) indicators of current disease activity;

 2) predictors of future disease progression; 

 3) predictors of future disease initiation at currently healthy sites. 

 Over 65 GCF components have been preliminarily examined as possible markers for the progression of periodontitis. These components fall into three general categories:

 • Host-derived enzymes and their inhibitors (**Table 1**);

 • Tissue breakdown products (**Table 2**).

 • Inflammatory mediators and host-response modifiers;

 The first two categories will be dealt in this Part, whereas, Part II will mainly contain Inflammatory mediators and host response modifiers i.e. category 3 and chair side point-of-care diagnostic aids.

**Table 1:** Host-derived enzymes and their inhibitors

Aspartate aminotransferase
Alkaline phosphatase
Acid phosphatase
β-Glucuronidase
Elastase
Elastase inhibitors
α2 - Macroglobulin
α1 - Proteinase inhibitor
Cathepsins
Cysteine proteinases (B, H, L)
Serine proteinase (G)
Cathepsin D
Trypsin-like enzymes
Immunoglobulin-degrading enzymes
Dipeptidyl peptidases
Nonspecific neutral proteinases
Collagenases
Matrix metalloproteinase-l (MMP-l)
Matrix metalloproteinase-3 (MMP-3)
Matrix metalloproteinase-8 (MMP-8)
Matrix metalloproteinase-13 (MMP-13)
Gelatinases
Matrix metalloproteinase-2 (MMP-2)
Matrix metalloproteinase-9 (MMP-9)
Tissue inhibitor of MMP-l (TIMP-l)
Stromyelysins
Myeloperoxidases
Lactate dehydrogenase
Arylsulfatase
β-N-acetyl-hexosaminidase

Aspartate aminotransferase – It is a cytoplasmic enzyme that is released upon cell death and elevated levels of total enzyme activity were found to be strongly associated with active disease sites [**10**]. Sites with severe gingival inflammation and progressive attachment loss demonstrate marked elevation in AST levels in GCF samples [**11**]. 

 Alkaline phosphatase - It is a membrane-based glycoprotein produced by many cells within the area of the periodontium and gingival crevice. The main sources of the enzyme are polymorphonuclear leukocytes (PMNs), gram-negative anaerobic bacteria associated with periodontal disease and osteoblast and fibroblast cells. Bacterial alkaline phosphatase (B-AP) aids in the uptake and metabolism of phosphorylated organic molecules, which bacteria require for growth and replication. The presence of B-AP is indicative of bacterial infection at the site. Alkaline phosphatase is thought to play a role in bone metabolism and mineralization and collagen formation. The activity of alkaline phosphatase has been show to be correlated with pocket depth and the percentage of bone loss [**12**] and this activity was found to be 20 times greater in GCF from active sites than in serum.

 Acid phosphatase - It has been widely investigated amongst the lysosomal enzymes and has often been used as a lysosomal marker. Quantitative analysis confirmed that gingival fluid contains 10-20 times more acid phosphatase than serum. The host sources are the PMNs and desquamating epithelial cells [**13**]. About 60% of the total acid phosphatase in whole gingival fluid originates from bacteria [**14**]. The levels of acid phosphatase do not correlate with measurements of disease severity or activity.

 β –Glucuronidase - It is one of the hydrolases found in the azurophilic or primary granules of PMNs [**13**]. The enzyme is liberated from macrophages, fibroblasts and endothelial cells of healthy or chronically inflamed gingival [**15**]. It is also positively associated with the number of Spirochetes, Porphyromonas gingivalis, Prevotella intermedia and lactose-negative black pigmenting bacteria in the subgingival flora. The level of β-glucuronidase correlates significantly with attachment loss that may subsequently occur in individuals with adult periodontitis [**16**].

 Elastase - Neutrophil elastase is a serine proteinase confined to the azurophil granules of PMNs which are analogous to lysozymes [**17**]. It acts upon elastin, proteoglycans, hemoglobin, fibrinogen and collagen. Leukocyte elastase degrades mature collagen fibers. Amounts of GCF elastase are greater in periodontitis patients than healthy controls [**18**].

 Elastase inhibitors - The activity of proteases in the tissues is probably modulated by the presence of inhibitors either produced locally or circulating in plasma. The main plasma inhibitors are α2-macroglobulin and α1-antitrypsin, which accounts for more than 90% of the total protease inhibiting capacity of serum. A third physiological inhibitor, α2-antichymotrypsin seems to inactivate only chymotrypsin-like enzymes, for instance Cathepsin G α2-macroglobulin inhibits all three neutral proteinases from PMN’s by a similar mechanism which consists of irreversible trapping of the enzyme molecule by the inhibitor. α1-antitrypsin inactivates mainly serine proteinases, elastase and cathepsin G and partially mammalian collagenase [**18**]. Both α1-antitrypsin and α2-macroglobulin were found in gingival fluid by Schenkein and Genco [**19**] in concentrations representing three-fourths of those found in serum. In inflamed gingiva, GCF samples had about twice as much α2- macroglobulin than the samples collected in the same area after therapy. 

 Cathepsins – It is an enzyme belonging to the class of cysteine proteinases. In GCF, macrophages are the main producers of cathepsin B [**20**]. GCF concentrations of cathepsin B were found to be elevated in patients with periodontal disease, but lower in patients with gingivitis [**21**]. Thus, it may have a potential use in distinguishing periodontitis from gingivitis and in planning treatment and monitoring treatment outcomes [**22**]. Cathepsin D, a carboxy endopeptidase, is present at high concentration in inflamed tissues. Its concentration is found to be 10 times higher in GCF during periodontal destruction [**23**]. Cathepsin G is serine endopeptidase contained in the azurophil granules of PMNs. It is also known as chymotrypsin like, because it attacks a number of synthetic substrates typical for chymotrypsin and is inhibited by the same inhibitors. It hydrolyzes hemoglobin and fibrinogen, casein, collagen and proteoglycans. Measurements of cathepsin and neutral proteases, have also shown a relationship to the severity of inflammation but no association with disease activity has been demonstrated [**24**]. 

Trypsin-like enzymes - Proteolythic activities associated with black-pigmented Bacteroides species have long been considered as virulence factors in the pathogenesis of periodontal disease [**25**]. Porphyromonas gingivalis, which is frequently isolated from periodontal lesions in adults with advanced periodontitis, possesses a spectrum of proteases including a trypsin-like enzyme [**26**]. The presence of this trypsin-like enzyme increases the potential of this organism to mediate destruction of periodontal tissues. It has to cleave peptide substrates with arginine terminal groups such as benzoyl-arginine-2 naphthylamide (BANA) or benzoyl-arginine-p-nitroanilide (BAPNA). The trypsin-like enzyme found in P. gingivalis is able to degrade collagen directly and its GCF levels might provide useful information on the periodontal condition [**27**].

 Immunoglobulin-degrading proteases – They constitute a group of microbial enzymes that have gained much interest due to their potential significance as virulence factors [**28**]. Such enzymes have been assumed to facilitate both bacterial colonization on mucous membranes and penetration of bacterial cells and their antigenic products through the mucosal barrier by elimination of immunoglobulins [**28**]. Consequently, immunoglobulin-degrading enzymes have been demonstrated mainly in pathogenic species and in species closely associated with infectious diseases [**28**]. GCF IgG antibodies to periodontopathic organisms are present in significantly higher levels in periodontal disease patients than in normal control subjects [**29**].

 Dipeptidyl Peptidases (DPP) - They are derived from lymphocytes, macrophages, and fibroblasts. DPP II has been localised to macrophages and fibroblasts30 in gingival tissue and in cells in GCF. DPP IV has been localised to monocytes, macrophages, fibroblasts and CD4 and CD8 lymphocytes [**30**]. They have the capacity to degrade collagen but their main function most likely lies in the activation of pro-forms of other enzymes, cytokines, and other immune mediators. Eley and Cox [**31**] monitored GCF levels of DPP II and IV and reported higher levels of both enzymes in sites with rapid and gradual attachment loss than in paired sites without attachment loss.

 Non-specific neutral proteases - Neutral protease is a non-specific metalloprotease. It cleaves fibronectin, collagen IV, and to a lesser extent collagen I, but it does not cleave collagen V or laminin. It hydrolyzes N-terminal peptide bonds of non-polar amino acid residues and may preferentially attack denatured and intercellular proteins with exposed hydrophobic amino acid residues [**32**]. It has been reported that an elevated level of neutral protease activity suggests an active phase of periodontal disease [**32**].

 Matrix Metalloproteinases - Host cell-derived enzymes such as matrix metalloproteinases (MMPs) are an important group of neutral proteinases implicated in the destructive process of periodontal disease that can be measured in GCF [**33**]. The neutrophils are the major cells responsible for MMP release at the infected site, specifically MMP-8 (collagenase-2) and MMP-9 (gelatinase-B) [**34**]. Although MMP-8 is able to potently degrade interstitial collagens, MMP-9 degrades several extracellular matrix proteins [**31**]. Mammalian collagenases initiate degradation by making a single cut; subsequent degradation of the denatured collagen molecule can be mediated by the gelatinases. GCF collagenase and collagenase activity has been shown to increase with increasing severity of inflammation and increasing pocket depth and alveolar bone loss [**35**]. Stromelysins (SL) are the major MMPs of fibroblast origin, and can activate fibroblast type collagenase [**36**]. Birkedal-Hansen et al. [**37**] have also suggested that SL may act as a marker of stromal cell involvement in the process of tissue degradation.

 TIMPs – They are locally produced and their main role is defending connective tissues in the very local area around the cell from which metalloproteinases are secreted. The tissue degradation is further thought to be induced by an imbalance between MMPs and TIMP [**38**] The mean amounts of SL and TIMP in diseased sites (gingivitis and periodontitis) is significantly higher than the mean amount of these GCF components in healthy sites [**39**].

 Myeloperoxidase - The myeloperoxidase-hydrogen peroxide-chloride system, which is part of the innate host defence mediated by polymorphonuclear leukocytes, possesses potent antimicrobial activity [**40**]. MPO is produced in the phagosome in excess concentrations of those that mediate bacterial killing. It has been suggested that MPO functions primarily to maintain a low concentration of hydrogen peroxide in the phagosome, thereby preserving the function of the granule proteases that otherwise would undergo irreversible oxidant-mediated inactivation if hydrogen peroxide accumulated in the phagosome [**41**]. High MPO levels in GCF from patients with progressive chronic periodontitis, and their reduction in response to treatment have been reported by Hernandez et al. [**42**].

Lactate dehydrogenase - It catalyses the reversible reduction of pyruvate to lactate. GCF contains 10-20 times more LDH than blood [**43**]. Although LDH is found in bacteria, most of its GCF concentration originates from the periodontal tissues. No significant correlation is found between the levels of LDH in gingival fluid and disease severity. As such it could reflect metabolic changes, such as the increase in anaerobic glycolysis characteristic of inflamed gingival [**44**].

 Arylsulfatase - It catalyzes the release of esterbound sulfate from a variety of O-sulfate esters and was shown to be higher in activity in GCF in gingivitis and periodontitis patients. Larnster and Co-workers [**45**] have examined the relationship between β-glucuronidase and arylsulfatase and have shown that levels in the GCF are elevated in inflamed relative to healthy non-inflamed sites, and that these levels decrease following periodontal treatment.

 β-N-acetyl-hexosaminidase (β-NAH) - It is an acid lysosomal hydrolase that emanates into GCF during neutrophilic phagocytosis. Under secretory conditions, the precursor forms of the newly synthesized enzyme will be liberated. During phagocytosis and cellular lysis, the lysosomal beta-N-acetyl-hexosaminidase is present. Untreated periodontitis is associated with elevated levels of myeloperoxidase, β-NAH, β-Glucoronidase, and Cathepsin D that may contribute to promoted loss of periodontal support and periodontal therapy induces a sustained down regulation of leukocyte activity as evidenced by the remission of GCF markers [**46**].

**Table 2:** Tissue breakdown products

Glycosaminoglycans
-Hyaluronic acid
-Chondroitin-4-sulfate
-Chondroitin-6-sulfate
-Dermatan sulfate
Hydroxyproline
Fibronectin fragments
Connective tissue and bone proteins
-Osteonectin
-Osteocalcin
-Type I collagen peptides
-Osteopontin
Laminin
Calprotectin
Hemoglobin β-chain peptides
Pyridinoline crosslinks (ICTP)
Polypeptide growth factors

 Glycosaminoglycans - Proteoglycans have a core protein on which one or more heteropolysaccharides (called glycosaminoglycans) are bound covalently. Different glycosaminoglycans can be found, depending on the tissue, although the most common are the nonsulfated hyaluronic acid, and the sulfated heparan sulfate, Dermatan sulphate, chondroitin-4 sulfate and chondroitin-6 sulfate. In general, chondroitin-4-sulfate is the most common glycosaminoglycan in the periodontium. Proteoglycans have the ability to bind most collagens as well as fibronectin. Upon degradation of periodontal tissues, glycosaminoglycans are released, making their way into the GCF. Chondroitin-4-sulfate appears to be the major glycosaminoglycan in untreated chronic periodontitis sites, as shown in both animal [**46**] and human [**47**] studies. Elevated glycosaminoglycan concentrations were also found in aggressive periodontal diseases, and associations have been made with periodontal pathogens such as P. gingivalis [**48**].

 Hydroxyproline - It is a characteristic amino acid is a major component of the collagen. Hydroxyproline and proline play key roles for collagen stability. They permit the sharp twisting of the collagen helix. Thus it is a major breakdown product of collagen present in the GCF [**49**].

 Fibronectin fragments - It is one of the components of the extracellular matrix (ECM) of periodontal tissue [**50**] its main role is in cell adhesion and proliferation, which explains its potential use in regenerative strategies. Cross-sectional studies have revealed that fibronectin is invariably found in a degraded form in the GCF, [**51,52**] and therefore is inactive [**53**]. Therefore, its presence in GCF would indicate FN fragmentation due to tissue destruction and not simply inflammation [**54**].

 Connective tissue and Bone proteins

 Osteonectin - Also referred to as secreted protein acidic and rich in cysteine and basement membrane protein (BM-40), osteonectin is a single-chain polypeptide that binds strongly to hydroxyapatite and other extracellular matrix proteins including collagens. Because of its affinity for collagen and hydroxylapatite, osteonectin has been implicated in the early phases of tissue mineralization [**55**]. In a cross-sectional study by Bowers et al.[**56**]. GCF samples were analyzed from patients with gingivitis, at moderate or severe periodontal disease states.

 Osteocalcin - It is a small calcium-binding protein of bone, and is the most abundant non collagenous protein of mineralized tissues [**57**]. Osteocalcin is predominantly synthesized by osteoblasts [**58**], and it has an important role in both bone resorption and mineralization [**59**]. Elevated serum osteocalcin levels have been shown in periods of rapid bone turnover [**60**]. Serum osteocalcin is presently considered a valid marker of bone turnover when resorption and formation are coupled, and a specific marker of bone formation when formation and resorption are uncoupled [**59**]. Relationship between GCF osteocalcin levels and periodontal disease have been reported [**61**].

 Type I collagen peptides - The most common extracellular matrix component is collagen, which is synthesized in a pro-form containing a terminal propeptide. After cleavage, these peptides are eliminated through the gingival pocket where they can be measured, thus they represent collagen biosynthesis and not degradation. Collagen I carboxy-terminal propeptide and collagen III amino-terminal propeptide were detectable in the GCF of patients with periodontitis, but not in healthy subjects, suggesting that turnover is higher in inflamed sites. The GCF levels of these collagens are increased after nonsurgical periodontal treatment, and return to baseline levels after a few days [**51,52**].

 Osteopontin (OPN) - It is a single-chain polypeptide. In bone matrix, OPN is highly concentrated at sites where osteoclasts are attached to the underlying mineral surface, that is, the clear zone attachment areas of the plasma membrane [**62**]. However, since OPN is produced by both osteoblasts and osteoclasts, it holds a dual function in bone maturation and mineralization as well as bone resorption [**63**] Sharma et al. [**64**] published findings from an investigation of GCF OPN that its concentrations increased proportionally with the progression of disease and when nonsurgical periodontal treatment was provided, its levels were significantly reduced.

 Laminin - It is a 900-kDa glycoprotein found in all basement membranes. During gingival inflammation, neutrophils leave the blood vessels and migrate through the connective tissue towards the inflammatory lesion, and some of them invade the gingival crevice. Steadman et al. [**65**] noted that a simple response against chemotactic factors seemed not to lead to basement membrane destruction, while activated neutrophils generated extensive destruction of the basement membrane. Higher amounts of laminin in GCF from patients with periodontitis suggest the presence of hyperactive neutrophils during the transmigration process through the endothelium/epithelium [**66**].

Calprotectin - It is a 36-kDa protein composed of a dimeric complex of 8- and 14-kDa subunits. Neutrophils are the primary source of calprotectin although other cells, such as activated monocytes and macrophages and specific epithelial cells, are also capable of manufacturing the protein. Calprotectin acts as a calcium- and zinc-binding protein with both antimicrobial and antifungal activities. It also plays a role in immune regulation through its ability to inhibit immunoglobulin production and acts as a proinflammatory protein for neutrophil recruitment and activation. In periodontology, Kido et al.[**67**] identified calprotectin in GCF and found that GCF concentration levels in patients with periodontal disease were higher than those in GCF from healthy subjects. The expression of calprotectin from inflammatory cells appears to offer protection of the epithelial cells against binding and invasion by P. gingivalis. In periodontal disease, it appears to improve resistance to P. gingivalis by boosting the barrier protection and innate immune functions of the gingival epithelium [**68**].

Hemoglobin β-chain peptides - 2 peptides derived from the hemoglobin (Hb) are β-chain decapeptide and a dodecapeptide. They are pharmacologically and physiologically active, and act as inflammatory mediators [**69**]. Both peptides may also act as substrates of proline-specific peptidases studied in treponemes isolated from the human subgingival dental plaque [69]. These two particular Hb β-chain sequences were present in GCF and successful periodontal therapy will reduce the levels of these peptides [**70**].

 Pyridinoline crosslinks (ICTP) – They represent a class of collagen-degrading molecules that include pyridinoline, deoxypyridinoline, N-telopeptides, and C-telopeptides [**71**]. Subsequent to osteoclastic bone resorption and collagen matrix degradation these molecules are released into the circulation. Given their specificity for bone resorption, pyridinoline cross-links represent a potentially valuable diagnostic aid in periodontics, because biochemical markers specific for bone degradation may be useful in differentiating the presence of gingival inflammation from active periodontal and peri-implant bone destruction [**72**].

 Polypeptide growth factors are a class of natural biological mediators that regulate key cellular events in tissue repair, including cell proliferation, chemotaxis, differentiation, and matrix synthesis, by binding to specific cell-surface receptors [**73**]. Several growth factors are concentrated in the organic matrix of bone and released during bone resorption [**74**], and are therefore suggested to play a role in bone remodelling through regulation of the coupling process of bone resorption and formation [**75**]. There are several studies in the periodontal literature examining GCF and salivary levels of growth factors for periodontal disease diagnosis including of epidermal growth factor (EGF),[**76**] transforming growth factor-α (TGF-α) and TGF-β,[**74**] platelet-derived growth factor (PDGF),[**77**] and vascular-endothelial growth factor (VEGF) [**78**].

## Conclusion

GCF is a vehicle for monitoring tissue and cell products and allows a degree of non-invasive access to the periodontium, unlike the majority of other tissues in the body. It carries multiple molecular factors derived from the host response and is considered a significant protective mechanism in periodontal infection. Substantial improvements have been made in the understanding of the mediators implicated in the initiation, pathogenesis, and progression of periodontitis. Evaluation of the markers in GCF is considered a good method in the determination of a person’s risk for periodontal disease.
